# RNA-sequencing profiling analysis of pericyte-derived extracellular vesicle–mimetic nanovesicles-regulated genes in primary cultured fibroblasts from normal and Peyronie’s disease penile tunica albuginea

**DOI:** 10.1186/s12894-021-00872-x

**Published:** 2021-08-06

**Authors:** Guo Nan Yin, Shuguang Piao, Zhiyong Liu, Lei Wang, Jiyeon Ock, Mi-Hye Kwon, Do-Kyun Kim, Yong Song Gho, Jun-Kyu Suh, Ji-Kan Ryu

**Affiliations:** 1 Department of Urology and National Research Center for Sexual Medicine, Inha University School of Medicine, 7-206, 3rd St, Shinheung-Dong, Jung-Gu, Incheon, 22332 Republic of Korea; 2 Department of Urology at Changhai Hospital Affiliated with the Naval Medicine University, Shanghai, 200433 People’s Republic of China; 3 Korea Zoonosis Research Institute, Jeonbuk National University, Iksan, Jeonbuk 54531 Korea; 4 Department of Life Sciences, Pohang University of Science and Technology, Pohang, Kyeongsangbuk-do 37673 Korea

**Keywords:** Peyronie’s disease, Pericytes, Nanovesicles, Gene expression, RNA-sequencing

## Abstract

**Background:**

Peyronie’s disease (PD) is a severe fibrotic disease of the tunica albuginea that causes penis curvature and leads to penile pain, deformity, and erectile dysfunction. The role of pericytes in the pathogenesis of fibrosis has recently been determined. Extracellular vesicle (EV)–mimetic nanovesicles (NVs) have attracted attention regarding intercellular communication between cells in the field of fibrosis. However, the global gene expression of pericyte-derived EV–mimetic NVs (PC–NVs) in regulating fibrosis remains unknown. Here, we used RNA-sequencing technology to investigate the potential target genes regulated by PC–NVs in primary fibroblasts derived from human PD plaque.

**Methods:**

Human primary fibroblasts derived from normal and PD patients was cultured and treated with cavernosum pericytes isolated extracellular vesicle (EV)–mimetic nanovesicles (NVs). A global gene expression RNA-sequencing assay was performed on normal fibroblasts, PD fibroblasts, and PD fibroblasts treated with PC–NVs. Reverse transcription polymerase chain reaction (RT-PCR) was used for sequencing data validation.

**Results:**

A total of 4135 genes showed significantly differential expression in the normal fibroblasts, PD fibroblasts, and PD fibroblasts treated with PC–NVs. However, only 91 contra-regulated genes were detected among the three libraries. Furthermore, 20 contra-regulated genes were selected and 11 showed consistent changes in the RNA-sequencing assay, which were validated by RT-PCR.

**Conclusion:**

The gene expression profiling results suggested that these validated genes may be good targets for understanding potential mechanisms and conducting molecular studies into PD.

**Supplementary Information:**

The online version contains supplementary material available at 10.1186/s12894-021-00872-x.

## Background

Peyronie’s disease (PD) is caused by excessive fibrosis and scar tissue formation in the tunica albuginea (TA), resulting in penile pain, abnormal curvature, and erectile dysfunction (ED) [1, 2]. Although the existence of PD has been known for a long time, the pathophysiology of PD has not been studied as widely as fibrosis in other organs, such as the kidneys, liver, or lungs. Currently, the most available medical therapy is collagenase and interferon injection and surgical intervention [3, 4]. However, these treatments can cause glandular hypoesthesia and a high risk of new onset ED [5]. Therefore, the identification of novel therapeutic targets related to PD fibrosis is required.

Pericytes play a fundamental role in vascular contractility and stability, regulation of vascular development, and as a storage vault of mesenchymal stem cells [6, 7]. In vitro studies have shown that pericytes exhibit fibrogenic potential [8, 9] and transition to myofibroblasts [10]. Moreover, the inhibition of angiogenesis may be effective in the suppression of fibrosis [11]. However, recently studies have shown that the inhibition of angiogenesis may aggravate fibrosis [12, 13]. These findings suggest that different antiangiogenic and molecular targets produce different results in the treatment of fibrosis. We recently reported in a mouse model of diabetic ED that pericyte-derived angiogenic factor restored erectile function by enhancing cavernous angiogenesis [14, 15].

Extracellular vesicles (EVs) were previously believed to be cell excretions. However, a number of studies have shown that EVs contain proteins, lipids, and RNA, which can affect the physiological and pathological communications between cells [16, 17]. Many studies regarding the potential role of EVs have been conducted for human diseases, including strokes [18], tumor metastasis [19], and kidney disease [20]. Therefore, clarifying the role of EVs in fibrosis would be beneficial to aid in the understanding of fibrosis mechanisms. However, one of the major limitations of EVs is the low production yield [21]. Therefore, to maximize the production of vesicles, we used a mini extruder system and extracted more than 100-fold greater EV–mimetic NVs from pericytes. The cell-derived EV–mimetic NVs showed similar characteristics to the natural EVs [22]. We recently demonstrated that intracavernous injection of PC–NVs induced penile angiogenesis and rescued erectile function in a mouse model of cavernous nerve injury [15]. Along with potential relationship between angiogenesis and suppression of tissue fibrosis [11–13], the angiogenic potential of pericytes and PC–NVs led us to investigate the potential role of PC–NVs in the fibrogenic process of PD.

Gene expression profiling analysis in physiological and pathological conditions can provide a foundation for studying the mechanisms of fibrosis in PD. In the present study, we performed an RNA-sequencing assay on normal fibroblasts, PD fibroblasts, and PD fibroblasts treated with PC–NVs.

## Methods

### Ethics statement and study design

All TA tissues and animals used in this study were approved by the Institutional Review Board (IRB No: 2007-730) and the Institutional Animal Care and Use Committee of our University (approval number: 171129-527), respectively. The plaque tissue of a patient with PD (48 years old) and the normal TA tissue from control patients (undergoing penoplasty for congenital curvature, 21 years old) were used for the human fibroblast culture study. In addition, 10 adult male C57BL/6J mice (8 weeks old, Orient Bio, Korea) were used for the mouse cavernous pericytes (MCPs) primary culture.

### Primary culture and characterization of human fibroblasts

The TA tissues were used for the primary fibroblast culture as described previously [23, 24]. Briefly, PD plaque and normal TA tissues were maintained in sterile vials with Hank’s balanced salt solution (HBSS, Gibco, Carlsbad, CA, USA) and washed three times with phosphate-buffered saline (PBS). The TA tissues were cut into 1–2 mm sections and incubated in 12.5 mL Dulbecco’s modified Eagle’s medium (DMEM, Gibco) supplemented with 0.06% collagenase A (Sigma-Aldrich, St. Louis, MO, USA) at 37 °C for 1 h in a 5% CO_2_ atmosphere. The cells and tissue fragments were collected by centrifugation (400 g for 5 min), washed with fresh culture medium, and placed in 100 mm cell culture dishes (Falcon-Becton Dickinson Labware, Franklin Lakes, NJ, USA) with DMEM containing 10% fetal bovine serum (FBS), penicillin (100 U/mL), and streptomycin (100 μg/mL) at 37 °C in a 5% CO_2_ atmosphere. Media were changed every 2 days and the cells were characterized as previously described [23, 24]. Passages 5 to 8 were used for the experiments.

To determine cell type, the cells were cultured on sterile cover glasses, placed into 12-well plates and grown until nearly confluent. The cells were stained with antibody to CD90 (fibroblast marker, R&D Systems Inc., Minneapolis, MN, USA; 1:100), Vimentin (fibroblast marker, R&D Systems Inc., 1:50), NG2 chondroitin sulfate proteoglycan (NG2, pericyte marker, Millipore, San Francisco, CA, USA; 1:50), CD31 (endothelial cell marker, Chemicon, Temecula, CA, USA; 1:50), or DAPI (nucleus marker; Vector Laboratories Inc., Burlingame, CA, USA), as previously described [25]. Signals were visualized and digital images were obtained using a confocal fluorescence microscope (K1-Fluo, Nanoscope Systems, Inc., Daejeon, Korea).

### Primary culture of MCPs

The primary cultures of MCPs were performed as described previously [26, 27]. Shortly, 8 weeks old male C57BL/6J mice were anesthetized with ketamine (100 mg/kg) and xylazine (5 mg/kg) intramuscularly, and sacrificed by cervical dislocation. Then, the penis tissues were harvested and maintained in sterile vials with HBSS (Gibco). After washing three times with PBS, the urethra and dorsal neurovascular bundle were removed, and only the corpus cavernosum tissues were used. The corpus cavernosum tissues were cut into approximately 1–2 mm sections and settled via gravity into collagen I-coated 35 mm cell culture dishes with 300 µL complement DMEM (GIBCO) at 37 °C for 20 min in a 5% CO_2_ atmosphere. Thereafter, 900 µL of complement medium was added and incubated at 37 °C with 5% CO_2._ The complement medium contained 20% FBS, 1% penicillin/streptomycin, and 10 nM human pigment epithelium-derived factor (PEDF; Sigma-Aldrich). The medium was changed every 2 days, and after approximately 10 days sprouting cells were sub-cultured into collagen I (Advanced BioMatrix, San Diego, CA, USA)-coated dishes. Cells from passages 2 to 3 were used for the experiments.

### Preparation and characterization of MCP-derived EV–mimetic nanovesicles (NVs)

MCP-derived EV–mimetic NVs (PC–NVs) were prepared using a mini extruder system (Avanti Polar Lipids, Birmingham, AL, USA), as described previously [28, 29]. Briefly, MCPs were washed three times with PBS, detached with 0.25% Trypsin–EDTA (Invitrogen, Carlsbad, CA, USA) and re-suspended in 4-(2-Hydroxyethyl)piperazine-1-ethanesulfonic acid (HEPES)buffer solution (Gibco). The cell suspension was sequentially extruded 10 times across 10, 5, and 1 μm pore-sized polycarbonate membranes (Nuclepore, Whatman Inc., Clifon, NJ, USA), respectively. Next, ultracentrifugation was performed at 100,000 g for 2 h at 4 °C with a step gradient, which was formed with 50% iodixanol (1 mL; Axis-Shield PoC AS, Oslo, Norway) overlaid with 10% iodixanol (2 mL) and topside with the extruded samples (7 mL). PC–NVs were filtered with a 0.45 μm filter and stored at − 80 °C until further use. To quantify the PC–NVs, the EXOCET exosome quantitation assay kit (System Biosciences, Palo Alto, CA, USA) was used, and 1 µg/µL of the final concentration of the PC–NVs was prepared for all experiments.

### Western blotting

For the immunoblot analyses of PC–NVs, equal protein amounts (10 µg) of purified PC–NVs and whole cells extracted using RIPA lysis buffer (Sigma-Aldrich) were separated by SDS-PAGE (12% gel) and transferred to polyvinylidene fluoride (PVDF) membranes. Each blot was blocked and incubated with antibodies to GM130 (BD Biosciences, San Jose, CA, USA; 1:1000), CD9 (Abcam, Cambridge, UK; 1:1000), CD81 (Novus Biologicals; 1:1000), or TSG101 (Novus Biologicals; 1:500).

### RNA-sequencing assay

For the RNA-sequencing study, the normal and PD TA-derived fibroblasts were cultured and treated with PC–NVs (n = 4 per group). The RNA-sequencing assay was performed by E-Biogen Inc. (Korea). Briefly, total RNA was isolated 24 h after exposure to PC–NVs using TRIzol reagent (Invitrogen). RNA quality was assessed using an Agilent 2100 Bioanalyzer (Agilent Technologies, Amstelveen, The Netherlands), and RNA quantification was performed using an ND-2000 Spectrophotometer (Thermo Inc., DE, USA).

### Library sequencing and data analysis

Libraries were prepared from total RNA using the SMARTer Stranded RNA-Seq Kit (Clontech Laboratories, Inc., USA). The isolation of mRNA was performed using the Poly(A) RNA Selection Kit (LEXOGEN, Inc., Austria). Indexing was performed using the Illumina indices 1–12. The enrichment step was performed using PCR. Subsequently, libraries were checked using the Agilent 2100 Bioanalyzer (DNA High Sensitivity Kit) to evaluate the mean fragment size. Quantification was performed using the library quantification kit using a StepOne Real-Time PCR System (Life Technologies, Inc., USA). High-throughput sequencing was performed as paired-end 100 sequencing using HiSeq 2500 (Illumina, Inc., USA).

Quality control of the raw sequencing data was performed using FastQC (https://www.bioinformatics.babraham.ac.uk/projects/fastqc/). Adapter and low-quality reads (< Q20) were removed using FASTX_Trimmer (http://hannonlab.cshl.edu/fastx_toolkit/) and BBMap (https://sourceforge.net/projects/bbmap/). Then, the trimmed reads were mapped to the reference genome using TopHat [30]. Gene expression levels were estimated using RC (read count) and FPKM (fragments per kb per million reads) values by BEDTools [31] and Cufflinks [32]. The expression values were normalized with the Quantile normalization method using EdgeR within R (https://www.r-project.org). Data mining and graphic visualization were performed using ExDEGA (E-Biogen, Inc., Korea). The datasets generated and/or analyzed during the current study are available in the Gene Expression Omnibus repository (www.ncbi.nlm.nih.gov/geo/query/acc.cgi?acc=GSE146500. Accession no. GSE146500).

### Validation of sequencing data by RT-PCR

Total RNA was extracted from cultured cells using TRIzol (Invitrogen) following the manufacturer’s protocols. Reverse transcription was performed using 1 µg of RNA in 20 µL of reaction buffer with oligo dT primer and AccuPower RT Premix (Bioneer Inc., Korea). The PCR reaction was performed with denaturation at 94 °C for 30 s, annealing at 60 °C for 30 s, and extension at 72 °C for 1 min in a DNA Engine Tetrad Peltier Thermal Cycler. For the analysis of PCR products, 10 µL of each PCR productwas electrophoresed on a 1% agarose gel and detected under ultraviolet light. GAPDH was used as an internal control [14].

All digital image, western blot, and PCR band densitometry analyses were performed using an image analyzer system (National Institutes of Health [NIH] ImageJ 1.34, http://rsbweb.nih.gov/ij/).

### Statistical analysis

All data are expressed as means ± standard errors. Statistical analysis was performed using Student t-test. *p* values less than 0.05 were considered statistically significant.

## Results

### Identification of human fibroblasts

**Fig. 1:**
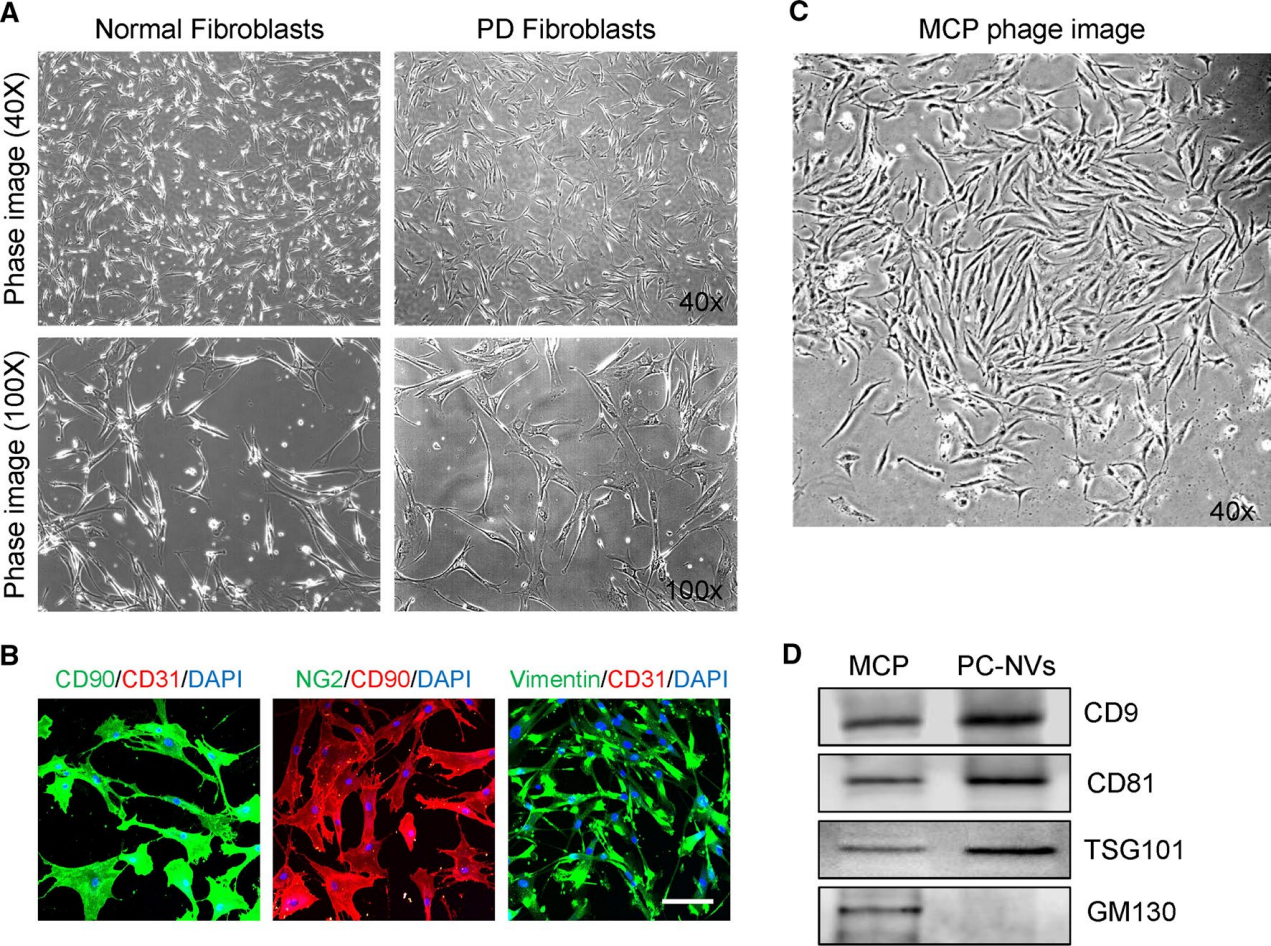
Isolation and characterization of fibroblasts from human tunica albuginea (TA) and extracellular vesicle (EV)–mimetic nanovesicles (NVs) from mouse cavernous pericytes (MCPs). **A** Representative phage images (screen magnification ×40 and ×100) of primary cultured fibroblasts from the TA tissues of healthy subjects and those with PD at passage 5. **B** Immunofluorescent staining of fibroblasts with antibodies against CD90 (fibroblast marker), Vimentin (fibroblast marker), NG2 (pericyte marker), and CD31 (endothelial cell marker). Nuclei were labeled with the DNA dye DAPI. Scale bar indicates 100 μm. DAPI = 4,6-diamidino-2-phenylindole. **C** Representative phage images (screen magnification ×40) of primary cultured mouse cavernous pericytes at passage 2. **D** Representative western blot for EV positive markers (CD9, CD81, and TSG101) or an EV negative marker (GM130) in MCPs and pericyte-derived EV–mimetic NVs

The fibroblasts were isolated from human normal and PD plaque tissues. Representative images showed high positive staining for CD90 and Vimentin (fibroblast markers) of more than 95%, but not for pericyte (NG2) or endothelial cell (CD31) markers (Fig. 1A, B).

### PC–NV preparation and characterization

PC–NVs were prepared from MCPs according to previous methods [28]. Western blot analysis showed that PC–NVs displayed positive exosomes markers, including CD9, CD81, and TSG101, but not for negative marker GM130 (Fig. 1C, D).

### Transcriptional profiling and gene ontology (GO) category analysis

**Fig. 2:**
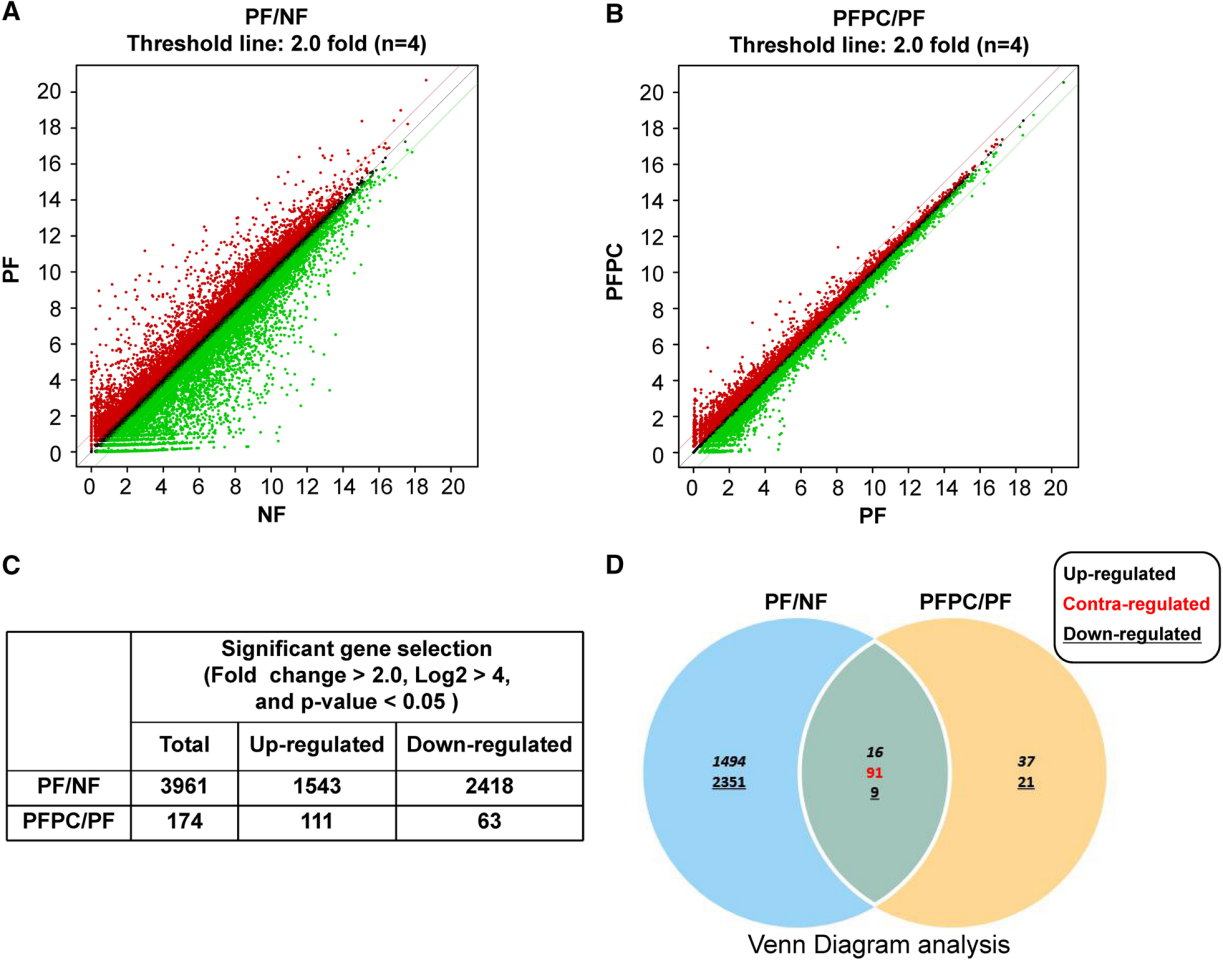
Analysis of differentially expressed genes (DEGs) from three libraries. **A** Total gene expression of normal fibroblasts (NF) and PD fibroblasts (PF). **B** Total gene expression of PF and PC–NV-treated PF (PFPC) groups. **C** DEG analysis for significantly altered gene selection according to the conditions set: fold-change > 2.0, log2 > 4, and *p* value < 0.05. **D** Venn diagram analysis between PF/NF and PFPC/PF

For this study, three gene libraries for the normal fibroblast (NF), PD fibroblast (PF), and PC–NVs-treated PF (PFPC) groups were constructed for an RNA-sequencing assay (n = 4 for each group). In total, 25,737 genes were detected in three libraries. Significant gene selection was performed with three conditions: fold-change > 2.0, log2 > 4, and *p* value < 0.05. Among all detected genes, 3961 showed significant differential expression in the PF group compared with the NF group, and 174 were significantly differentially expressed in the PFPC group compared with the PF group (Fig. 2A–C). Only 91 contra-regulated genes (Additional file 1: Table S1) were detected between PF/NF and PFPC/PF through Venn diagram analysis (Fig. 2D).

**Fig. 3:**
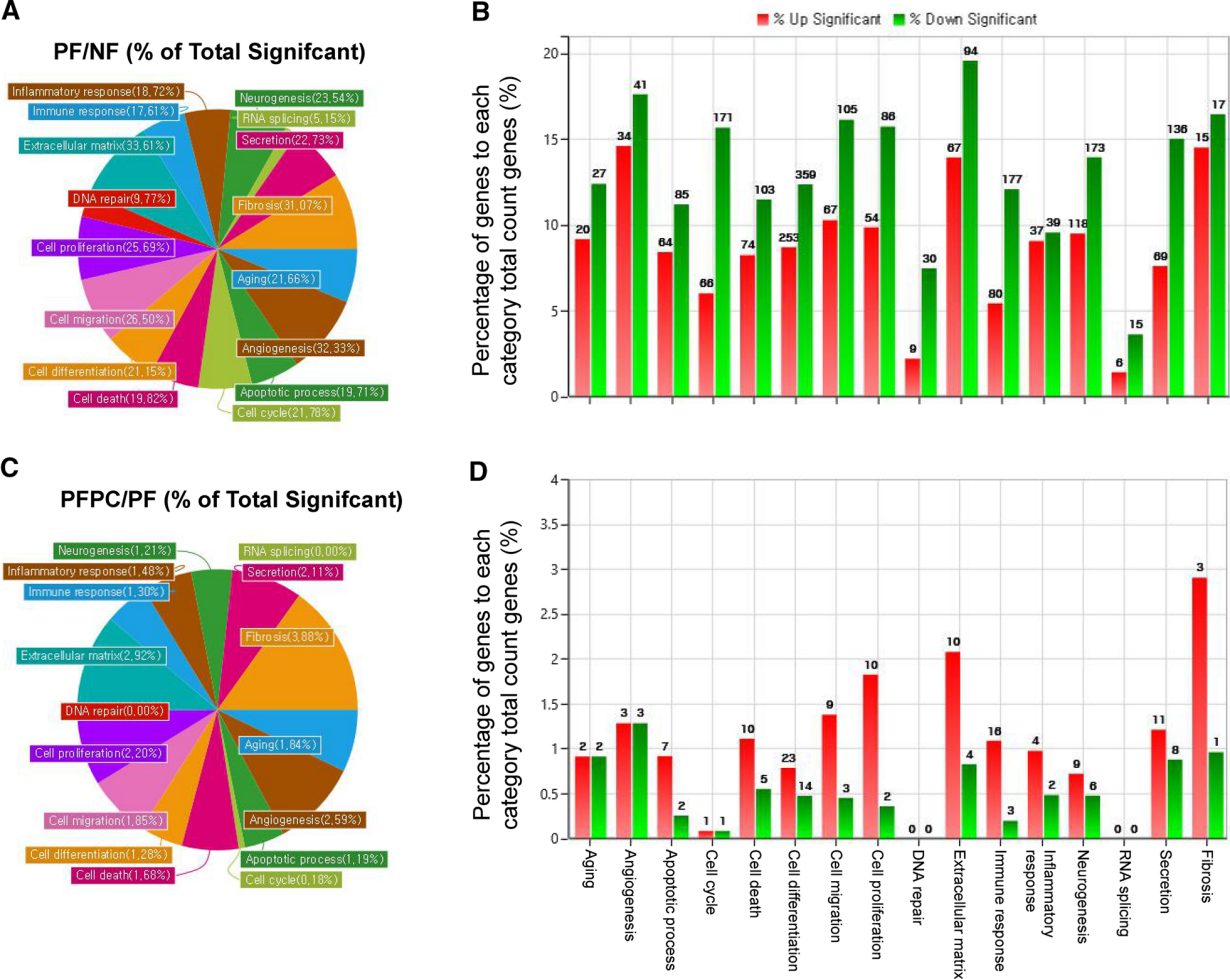
Significantly differentially expressed genes (DEGs) of the RNA-sequencing data were distributed to gene ontology (GO) categories. **A**, **B** The distribution (**A** total percentage; **B** detailed percentage and numbers of upregulated and downregulated genes, respectively) of total significantly DEGs in 16 GO categories in the PD fibroblast (PF) group compared with the normal fibroblast (NF) group. **C**, **D** The distribution (**C** total percentage; **D** detailed percentage and numbers of upregulated and downregulated genes, respectively) of total significantly DEGs in 16 GO categories in the PC–NV-treated PF group compared with the PF group

To evaluate the GO categories, all significantly differentially expressed genes (DEGs) were classified in 16 GO categories by ExDEGA software (E-Biogen, Inc., Korea). Among these, extracellular matrix (33.61%), angiogenesis (32.33%), and fibrosis (31.07%) made up the largest proportion in the PF group compared with the NF group (Fig. 3A, B). However, fibrosis (3.88%), extracellular matrix (2.92%), and angiogenesis (2.59%) made up the largest proportion in the PFPC group compared with the PF group (Fig. 3C, D).

### Validation of RNA-sequencing results by RT-PCR

**Fig. 4:**
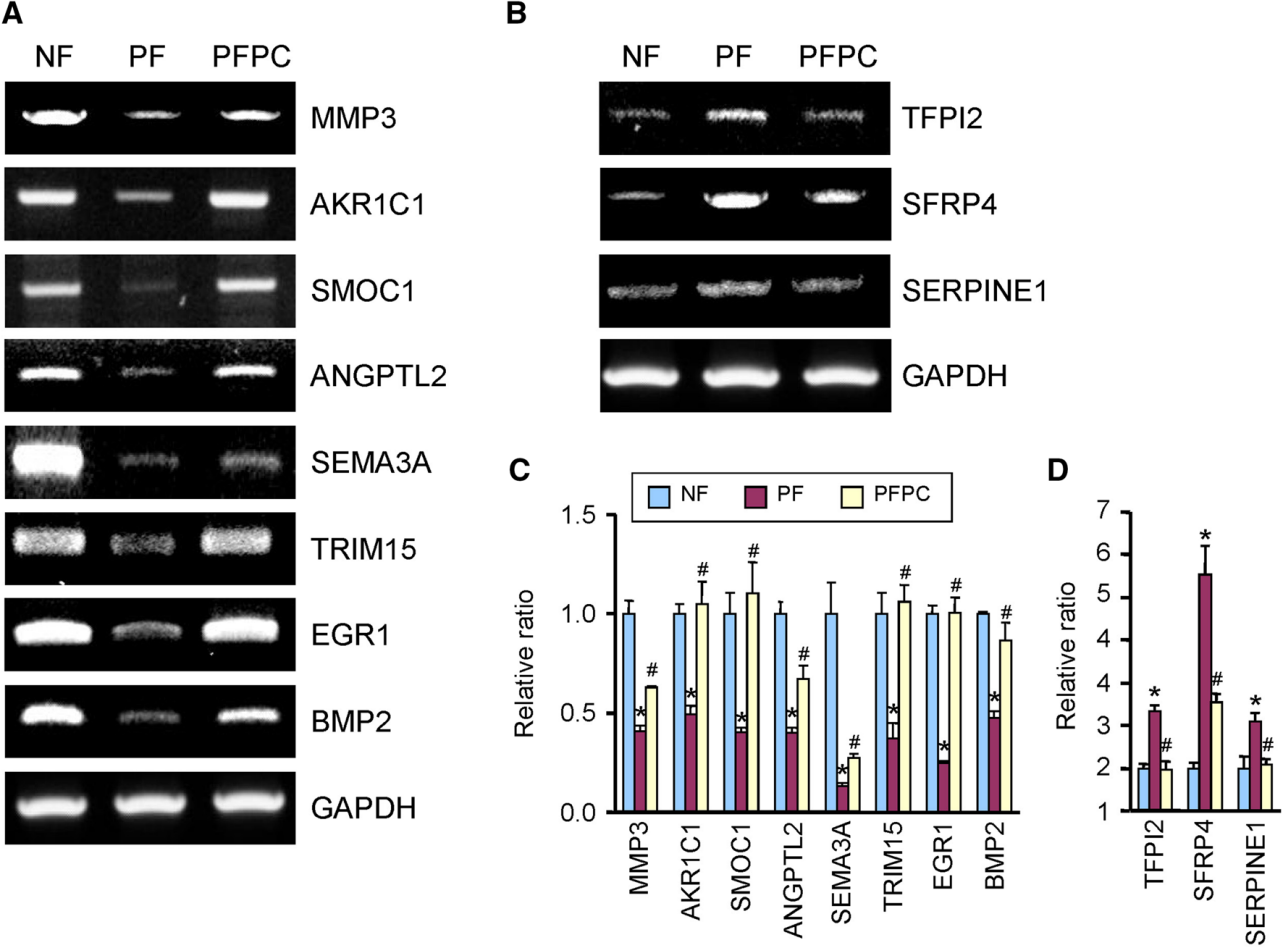
RT-PCR validation of differentially expressed genes selected from the RNA-sequencing assay. **A**, **B** Eleven contra-regulated genes were found to be consistent with the RNA-sequencing result in normal fibroblast (NF), PD fibroblast (PF), and PC–NV-treated PF (PFPC) groups. **C**, **D** Each bar depicts the mean value (± standard error) from three separate experiments. *p < 0.001 compared with the NF group. ^#^*p* < 0.05 compared with the PF group

To validate the RNA-sequencing results, we selected 20 genes (Additional file 1: Table S2) from 91 contra-regulated DEGs, and 11 (primers as shown in Additional file 1: Table S3) showed results consistent with the RNA-sequencing assay by RT-PCR. Among these genes, *MMP3, AKR1C1, SMOC1, ANGPTL2, SEMA3A, TRIM15, EGR1,* and *BMP2* were downregulated in the PF group compared with the NF group, and were significantly recovered in the PFPC group (Fig. 4A, C). Only *TFPI2, SFRP4*, and *SERPINE1* were induced in the PF group and recovered in the PFPC group (Fig. 4B, D).

## Discussion

The accurate physiological and pathological mechanisms of PD remain poorly understood. To date, most gene expression studies have focused on human PD plaque at a tissue level in vivo [33, 34] but not at a cellular level in vitro*.* Therefore, to investigate the exact mechanisms and potential target genes for PD, we cultured human fibroblasts from human PD plaque and performed RNA-sequencing assays.

EVs display a potential role in kidney fibrosis and other fibrotic diseases [20, 35]; however, little is currently known regarding the detailed mechanisms. Considering the low yield of EVs, we extracted more than 100-fold greater EV–mimetic NVs from MCPs, which were primarily cultured from mouse corpus cavernosum tissues. Many previous studies have found that pericytes display diverse features in relation to fibrosis that are dependent on different molecular targets [8, 9, 11]. We used pericytes isolated from mouse cavernous tissue, but not a human tissue, because it was not easy to harvest human erectile tissue in a large quantity. Although there are animal models of PD by using intratunical injection of fibrin [36] or transforming growth factor-β1 [37, 38], these animal models cannot represent the complex pathophysiologic processes of the human PD. Meanwhile, we can easily obtain fibrotic plaque from the patients with PD during reconstructive surgery. In the present study, therefore, human PD fibroblasts exposed to mouse origin of PC–NVs were compared with human PD fibroblasts to investigate the regulation of gene expression by PC–NVs in PD.

From the RNA-sequencing assay, 3961 DEGs were detected, and the 16 top GO categories were assessed in this study. GO analysis showed that significantly altered genes were enriched in the extracellular matrix, angiogenesis, and fibrosis. The extracellular matrix is a driver of progressive fibrosis [39], and angiogenesis is closely associated with chronic liver fibrosis [20]. These data suggest that our DEG detection is credible. However, the molecular basis of PC–NVs in regulating the extracellular matrix or angiogenesis pathway in PD remains largely unknown. In this study, only 91 contra-regulated genes were identified from the three libraries (NF, PF, and PFPC). After precision screening, 20 genes were selected and validated by RT-PCR in same conditions. However, only 11 genes were validated to be consistent with the RNA-sequencing results as showed in Fig. 4. Through literature analysis, we found that most target genes have multiple roles, such as angiogenesis*,* extracellular matrix (ECM), and fibrosis. Some studies have shown that the *MMP3* attenuated the process of fibrosis by degrading α2-antiplasmin and ECM [40] and these enzymes inhibitors are up-regulated (*TFPI2*) [41]. Many studies have shown that fibrosis and angiogenesis have common characteristics. Wound healing is a typical example of the synergistic interaction between the molecular mechanisms of fibrosis and angiogenesis [42, 43]. Although some studies have shown that *AKR1C1* [44]*, SMOC1* [45]*, ANGPTL2* [46]*, SEMA3A* [47]*, TRIM15* [48]*, EGR1* [49]*, BMP2* [50]*, SFRP4* [51]*,* and *SERPINE1* [52] are related to angiogenesis or fibrosis, but the detailed mechanism of action of these genes is still unknown, and it will be interesting to study the network and pathway of these genes in fibrosis and angiogenesis in the future. Therefore, on the one hand, we hypothesized that pericytes might be releasing nanovesicles, which contain these target genes to directly affect nearby fibroblasts, such as increase angiogenesis and reducing excessive pathological ECM deposition during fibrosis. On the other hand, pericyte-derived nanovesicles might indirectly affect the expression of these target genes in fibroblasts through microRNA, and further research is needed to resolve these hypotheses. These genes may be the key to understanding how PC–NVs regulate the extracellular matrix, angiogenesis, and fibrosis mechanisms in PD.

To the best of our knowledge, this is the first study to demonstrate the systematic profiling of gene alterations in NF, PF, and PFPC. However, the present study has some limitations. First, a small number of cultured human fibroblast samples were used in target gene validation and age differences existed among groups, and need to collect more age-matched samples for further studies. It would be more appropriate to use healthy TA from an age-matched PD patient instead of using TA from a young patient with congenital penile curvature. Second, we were unable to demonstrate the network of these validated genes in the extracellular matrix, angiogenesis, and fibrosis pathways. Further studies are needed to document the function of each gene at cellular and molecular levels as well as in animal models of PD. Third, mouse corpus cavernous pericytes were used for EV–mimetic NVs isolation, and further research is needed to cultivate human corpus cavernous pericytes and isolate human PC–NVs and process them in human PD fibroblasts to study the detailed mechanism of these initial selected target genes.

## Conclusion

In summary, we profiled the DEGs of human TA cultured fibroblasts in NF, PF, and PFPC groups. We hypothesize that these validated genes are good candidates for the study of the mechanism of PC–NVs in PD. Further studies exploring the effect of these target genes will be beneficial to further our understanding of the detailed mechanisms of the extracellular matrix, angiogenesis, and fibrosis in PD.

## Supplementary Information

**Additional file 1.** Supplementary tables for contra-regulated genes, primers for RT-PCR and un-cutted membrane or gels images.

## Data Availability

The datasets used during the current study available from the corresponding author on request.

## Abbreviations

PD: Peyronie’s disease
EV: Extracellular vesicle
NVs: Nanovesicles
PC–NVs: Pericyte-derived EV–mimetic NVs
RT-PCR: Reverse transcription polymerase chain reaction
TA: Tunica albuginea
ED: Rrectile dysfunction
MCPs: Mouse cavernous pericytes
HBSS: Hank’s balanced salt solution
PBS: Phosphate-buffered saline
DMEM: Dulbecco’s modified Eagle’s medium
FBS: Fetal bovine serum
PEDF: Pigment epithelium-derived factor
HEPES: 4-(2-Hydroxyethyl)piperazine-1-ethanesulfonic acid
PVDF: Polyvinylidene fluoride
