# Supplementary material for: RNA-sequencing profiling analysis of pericyte-derived extracellular vesicle–mimetic nanovesicles-regulated genes in primary cultured fibroblasts from normal and Peyronie’s disease penile tunica albuginea
Source: BMC Urol. 2021 Aug 6;21:103. doi: 10.1186/s12894-021-00872-x (PMC8344132; doi:10.1186/s12894-021-00872-x)
Supplement: Supplementary file 1 — Additional file 1. Supplementary tables for contra-regulated genes, primers for RT-PCR and un-cutted membrane or gels images. [file 12894_2021_872_MOESM1_ESM.docx]

Supplemental Materials

**RNA-sequencing profiling analysis of pericyte-derived extracellular vesicle-mimetic nanovesicles-regulated genes in primary cultured fibroblasts from normal and Peyronie’s disease penile tunica albuginea**

Guo Nan Yin^a,1^, Shuguang Piao^b,1^, Zhiyong Liu^b^, LeiWang^b^, Jiyeon Ock^a^, Mi-Hye Kwon^a^, Do-Kyun Kim^c^, Yong Song Gho^d^, Jun-Kyu Suh^a,^*, Ji-Kan Ryu^a,^*

^a^Department of Urology and National Research Center for Sexual Medicine, Inha University School of Medicine, Incheon 22332, Korea.

^b^Department of Urology at Changhai Hospital Affiliated with the Naval Medicine University, Shanghai 200433, People’s Republic of China.

^c^Korea Zoonosis Research Institute, Jeonbuk National University, lksan, Jeonbuk, 54531, Korea.

^d^Department of Life Sciences, Pohang University of Science and Technology, Pohang, Kyeongsangbuk-do 37673, Korea.

^1^These authors contributed equally to this work.

*Correspondence: Jun-Kyu Suh, MD, PhD and Ji-Kan Ryu, MD, PhD

Jun-Kyu Suh, MD, PhD

National Research Center for Sexual Medicine and Department of Urology

Inha University School of Medicine

7-206, 3^rd^ St, Shinheung-Dong, Jung-Gu, Incheon 22332

Republic of Korea

Tel: 82-32-890-3441, Fax: 82-32-890-3097

E-mail: jksuh@inha.ac.kr

Ji-Kan Ryu, MD, PhD

National Research Center for Sexual Medicine and Department of Urology

Inha University School of Medicine

7-206, 3^rd^ ST, Shinheung-Dong, Jung-Gu, Incheon 22332

Republic of Korea

Tel: 82-32-890-3505; Fax: 82-32-890-3099

E-mail: rjk0929@inha.ac.kr

Supplementary Table 1. List of Contra-regulated genes from RNA-sequencing assay

|  | **Ratio** | | | **Raw Data (RC)** | | |  |
| --- | --- | --- | --- | --- | --- | --- | --- |
| **Gene symbol** | **PT/ Con** | **PTPC/ Con** | **PTPC/ PT** | **Con** | **PT** | **PTPC** | **Description** |
| ADGRD1 | 198.904 | 64.987 | 0.327 | 1 | 239 | 77 | adhesion G protein-coupled receptor D1 |
| IL6 | 136.379 | 64.204 | 0.471 | 6 | 653 | 301 | interleukin 6 |
| PTPRB | 62.278 | 12.118 | 0.195 | 6 | 305 | 57 | protein tyrosine phosphatase, receptor type B |
| CEMIP | 44.378 | 6.800 | 0.153 | 49 | 1555 | 231 | cell migration inducing hyaluronan binding protein |
| OXTR | 26.730 | 10.788 | 0.404 | 49 | 952 | 376 | oxytocin receptor |
| IGFBP3 | 24.597 | 9.893 | 0.402 | 2338 | 41497 | 16369 | insulin-like growth factor binding protein 3 |
| LACAT8 | 21.751 | 5.116 | 0.235 | 0 | 19 | 4 | lung adenocarcinoma-associated transcript 8 |
| OLR1 | 18.915 | 6.191 | 0.327 | 4 | 70 | 21 | oxidized low density lipoprotein receptor 1 |
| EDN1 | 15.523 | 7.440 | 0.479 | 10 | 119 | 57 | endothelin 1 |
| ANKRD1 | 15.064 | 3.622 | 0.240 | 26 | 282 | 66 | ankyrin repeat domain 1 |
| FLG | 13.669 | 6.168 | 0.451 | 71 | 727 | 325 | filaggrin |
| SPTBN5 | 12.815 | 6.396 | 0.499 | 11 | 107 | 51 | spectrin beta, non-erythrocytic 5 |
| CD200 | 11.512 | 4.794 | 0.416 | 2 | 27 | 10 | CD200 molecule |
| KRT18 | 10.790 | 3.463 | 0.321 | 56 | 462 | 145 | keratin 18 |
| NPAS1 | 9.008 | 2.905 | 0.322 | 20 | 136 | 42 | neuronal PAS domain protein 1 |
| PTPRR | 8.624 | 2.111 | 0.245 | 1 | 13 | 3 | protein tyrosine phosphatase, receptor type R |
| HAPLN1 | 8.240 | 3.502 | 0.425 | 248 | 1470 | 611 | hyaluronan and proteoglycan link protein 1 |
| PKP2 | 7.750 | 3.404 | 0.439 | 7 | 46 | 19 | plakophilin 2 |
| SERPINE1 | 7.499 | 3.075 | 0.410 | 12274 | 66185 | 26542 | serpin family E member 1 |
| EXTL1 | 7.159 | 1.836 | 0.256 | 6 | 35 | 8 | exostosin-like glycosyltransferase 1 |
| MAMDC4 | 5.712 | 2.504 | 0.438 | 21 | 88 | 37 | MAM domain containing 4 |
| RIMS1 | 5.373 | 1.539 | 0.286 | 32 | 126 | 35 | regulating synaptic membrane exocytosis 1 |
| NRXN3 | 5.027 | 1.884 | 0.375 | 142 | 520 | 192 | neurexin 3 |
| CNN1 | 4.810 | 1.735 | 0.361 | 49 | 175 | 61 | calponin 1 |
| SYT12 | 4.715 | 2.178 | 0.462 | 5 | 18 | 8 | synaptotagmin 12 |
| ALPL | 3.683 | 1.697 | 0.461 | 16 | 45 | 20 | alkaline phosphatase, liver/bone/kidney |
| JPH2 | 3.389 | 1.411 | 0.416 | 23 | 58 | 23 | junctophilin 2 |
| KIRREL3-AS2 | 3.181 | 1.411 | 0.444 | 14 | 34 | 14 | KIRREL3 antisense RNA 2 |
| NR4A1 | 2.936 | 0.653 | 0.222 | 67 | 141 | 30 | nuclear receptor subfamily 4 group A member 1 |
| SFRP4 | 2.893 | 1.135 | 0.392 | 229 | 484 | 185 | secreted frizzled related protein 4 |
| TGM1 | 2.549 | 1.261 | 0.495 | 9 | 17 | 8 | transglutaminase 1 |
| OR2A1 | 2.385 | 0.984 | 0.413 | 13 | 24 | 9 | olfactory receptor family 2 subfamily A member 1 |
| METTL24 | 2.094 | 0.950 | 0.454 | 9 | 13 | 5 | methyltransferase like 24 |
| CKMT2 | 0.371 | 0.765 | 2.061 | 69 | 17 | 37 | creatine kinase, mitochondrial 2 |
| TFPI2 | 0.348 | 0.857 | 2.461 | 866 | 218 | 523 | tissue factor pathway inhibitor 2 |
| ACYP1 | 0.332 | 0.727 | 2.187 | 204 | 49 | 106 | acylphosphatase 1 |
| JUP | 0.332 | 0.800 | 2.409 | 65 | 15 | 36 | junction plakoglobin |

Supplementary Table 1. List of Contra-regulated genes from RNA-sequencing assay (Continued)

|  | **Ratio** | | | **Raw Data (RC)** | | |  |
| --- | --- | --- | --- | --- | --- | --- | --- |
| **Gene symbol** | **PT/ Con** | **PTPC/ Con** | **PTPC/ PT** | **Con** | **PT** | **PTPC** | **Description** |
| LOC100506801 | 0.328 | 0.726 | 2.213 | 80 | 18 | 40 | uncharacterized LOC100506801 |
| SIRT4 | 0.325 | 0.727 | 2.236 | 25 | 5 | 13 | sirtuin 4 |
| PDE4B | 0.322 | 1.613 | 5.008 | 116 | 26 | 131 | phosphodiesterase 4B |
| RNF144A | 0.318 | 1.089 | 3.428 | 319 | 72 | 243 | ring finger protein 144A |
| FAM87B | 0.318 | 0.652 | 2.052 | 37 | 8 | 17 | family with sequence similarity 87 member B |
| PLB1 | 0.317 | 0.671 | 2.115 | 41 | 9 | 20 | phospholipase B1 |
| OLFML2B | 0.296 | 0.693 | 2.340 | 1268 | 268 | 615 | olfactomedin like 2B |
| MMP10 | 0.278 | 0.892 | 3.208 | 28 | 5 | 18 | matrix metallopeptidase 10 |
| HTRA3 | 0.271 | 0.569 | 2.095 | 138 | 26 | 55 | HtrA serine peptidase 3 |
| BMP2 | 0.248 | 0.632 | 2.551 | 132 | 23 | 58 | bone morphogenetic protein 2 |
| TMEM158 | 0.221 | 0.596 | 2.699 | 80 | 12 | 33 | transmembrane protein 158 (gene/pseudogene) |
| ZNRD1 | 0.206 | 0.599 | 2.903 | 1446 | 224 | 624 | zinc ribbon domain containing 1 |
| SULT1A2 | 0.205 | 0.455 | 2.226 | 58 | 8 | 19 | sulfotransferase family 1A member 2 |
| EGR1 | 0.199 | 0.421 | 2.117 | 590 | 84 | 174 | early growth response 1 |
| PDE7B | 0.198 | 0.472 | 2.384 | 274 | 37 | 89 | phosphodiesterase 7B |
| DUSP4 | 0.196 | 0.618 | 3.156 | 198 | 27 | 86 | dual specificity phosphatase 4 |
| ADAM22 | 0.191 | 0.459 | 2.400 | 108 | 14 | 34 | ADAM metallopeptidase domain 22 |
| B3GNT5 | 0.191 | 0.460 | 2.406 | 127 | 17 | 41 | UDP-GlcNAc:betaGal beta-1,3-N-acetylglucosaminyltransferase 5 |
| SCARA3 | 0.184 | 0.429 | 2.331 | 6088 | 799 | 1825 | scavenger receptor class A member 3 |
| MAFB | 0.160 | 0.360 | 2.256 | 89 | 9 | 22 | MAF bZIP transcription factor B |
| HLA-J | 0.160 | 0.593 | 3.715 | 1682 | 204 | 722 | major histocompatibility complex, class I, J (pseudogene) |
| TRIM31-AS1 | 0.147 | 0.571 | 3.894 | 986 | 110 | 407 | TRIM31 antisense RNA 1 |
| TRIM15 | 0.147 | 0.573 | 3.907 | 1721 | 193 | 713 | tripartite motif containing 15 |
| TRIM40 | 0.146 | 0.572 | 3.907 | 1721 | 193 | 712 | tripartite motif containing 40 |
| CLU | 0.137 | 0.285 | 2.077 | 51857 | 5173 | 10508 | clusterin |
| TMEM86A | 0.137 | 0.320 | 2.337 | 65 | 6 | 14 | transmembrane protein 86A |
| YPEL4 | 0.130 | 0.307 | 2.358 | 92 | 8 | 20 | yippee like 4 |
| SEMA3A | 0.108 | 0.228 | 2.113 | 2617 | 197 | 413 | semaphorin 3A |
| RGS17 | 0.102 | 0.213 | 2.081 | 357 | 25 | 53 | regulator of G-protein signaling 17 |
| AGPAT9 | 0.101 | 0.209 | 2.068 | 182 | 12 | 26 | glycerol-3-phosphate acyltransferase 3 |
| DOCK4 | 0.096 | 0.204 | 2.134 | 1557 | 103 | 217 | dedicator of cytokinesis 4 |
| SLC1A3 | 0.095 | 0.203 | 2.133 | 154 | 10 | 21 | solute carrier family 1 member 3 |
| FBXO2 | 0.091 | 0.195 | 2.130 | 202 | 13 | 28 | F-box protein 2 |
| PTPRN | 0.084 | 0.212 | 2.538 | 516 | 31 | 78 | protein tyrosine phosphatase, receptor type N |
| RASL12 | 0.078 | 0.189 | 2.433 | 136 | 7 | 17 | RAS like family 12 |

Supplementary Table 1. List of Contra-regulated genes from RNA-sequencing assay (Continued)

|  | **Ratio** | | | **Raw Data (RC)** | | |  |
| --- | --- | --- | --- | --- | --- | --- | --- |
| **Gene symbol** | **PT/ Con** | **PTPC/ Con** | **PTPC/ PT** | **Con** | **PT** | **PTPC** | **Description** |
| FAM20A | 0.075 | 0.209 | 2.779 | 759 | 39 | 109 | family with sequence similarity 20 member A |
| PCSK1 | 0.075 | 0.159 | 2.128 | 181 | 9 | 19 | proprotein convertase subtilisin/kexin type 1 |
| CIT | 0.071 | 0.271 | 3.839 | 3081 | 152 | 573 | citron rho-interacting serine/threonine kinase |
| ANGPTL2 | 0.067 | 0.143 | 2.133 | 4825 | 231 | 484 | angiopoietin like 2 |
| SMOC1 | 0.064 | 0.220 | 3.434 | 806 | 37 | 122 | SPARC related modular calcium binding 1 |
| DENND2A | 0.062 | 0.149 | 2.391 | 436 | 19 | 46 | DENN domain containing 2A |
| VAV3 | 0.057 | 0.162 | 2.814 | 130 | 5 | 14 | vav guanine nucleotide exchange factor 3 |
| BTBD11 | 0.048 | 0.193 | 4.040 | 159 | 5 | 21 | BTB domain containing 11 |
| SPHKAP | 0.047 | 0.279 | 5.967 | 173 | 5 | 33 | SPHK1 interactor, AKAP domain containing |
| CCDC102B | 0.045 | 0.141 | 3.150 | 411 | 12 | 40 | coiled-coil domain containing 102B |
| RAB27B | 0.039 | 0.101 | 2.603 | 3756 | 101 | 260 | RAB27B, member RAS oncogene family |
| AKR1C1 | 0.039 | 0.078 | 2.031 | 30129 | 844 | 1675 | aldo-keto reductase family 1, member C1 |
| MMP3 | 0.036 | 0.102 | 2.812 | 1447 | 38 | 106 | matrix metallopeptidase 3 |
| SNCA | 0.034 | 0.091 | 2.642 | 525 | 12 | 34 | synuclein, alpha (non A4 component of amyloid precursor) |
| IL13RA2 | 0.015 | 0.037 | 2.414 | 711 | 7 | 18 | interleukin 13 receptor, alpha 2 |
| ALDH1A1 | 0.013 | 0.030 | 2.261 | 968 | 8 | 19 | aldehyde dehydrogenase 1 family member A1 |
| CLSTN2 | 0.011 | 0.027 | 2.426 | 1116 | 8 | 20 | calsyntenin 2 |
| ALDH3A1 | 0.002 | 0.007 | 3.721 | 3829 | 4 | 18 | aldehyde dehydrogenase 3 family member A1 |
| CHI3L1 | 0.001 | 0.002 | 2.080 | 11681 | 8 | 18 | chitinase 3 like 1 |

Supplementary Table 2. List of Contra-regulated genes validated by RT-PCR

|  | **Ratio** | | | **Raw Data (RC)** | | |  |
| --- | --- | --- | --- | --- | --- | --- | --- |
| **Gene symbol** | **PT/ Con** | **PTPC/ Con** | **PTPC/ PT** | **Con** | **PT** | **PTPC** | **Description** |
| MMP3 | 0.04 | 0.10 | 2.81 | 1447 | 38 | 106 | matrix metallopeptidase 3 |
| AKR1C1 | 0.04 | 0.08 | 2.03 | 30129 | 844 | 1675 | aldo-keto reductase family 1, member C1 |
| SMOC1 | 0.06 | 0.22 | 3.43 | 806 | 37 | 122 | SPARC related modular calcium binding 1 |
| ANGPTL2 | 0.07 | 0.14 | 2.13 | 4825 | 231 | 484 | angiopoietin like 2 |
| CIT | 0.07 | 0.27 | 3.84 | 3081 | 152 | 573 | citron rho-interacting serine/threonine kinase |
| DOCK4 | 0.10 | 0.20 | 2.13 | 1557 | 103 | 217 | dedicator of cytokinesis 4 |
| SEMA3A | 0.11 | 0.23 | 2.11 | 2617 | 197 | 413 | semaphorin 3A |
| CLU | 0.14 | 0.28 | 2.08 | 51857 | 5173 | 10508 | clusterin |
| TRIM15 | 0.15 | 0.57 | 3.91 | 1721 | 193 | 713 | tripartite motif containing 15 |
| EGR1 | 0.20 | 0.42 | 2.12 | 590 | 84 | 174 | early growth response 1 |
| BMP2 | 0.25 | 0.63 | 2.55 | 132 | 23 | 58 | bone morphogenetic protein 2 |
| OLFML2B | 0.30 | 0.69 | 2.34 | 1268 | 268 | 615 | olfactomedin like 2B |
| TFPI2 | 0.35 | 0.86 | 2.46 | 866 | 218 | 523 | tissue factor pathway inhibitor 2 |
| SFRP4 | 2.89 | 1.13 | 0.39 | 229 | 484 | 185 | secreted frizzled related protein 4 |
| NRXN3 | 5.03 | 1.88 | 0.38 | 142 | 520 | 192 | neurexin 3 |
| SERPINE1 | 7.50 | 3.08 | 0.41 | 12274 | 66185 | 26542 | serpin family E member 1 |
| HAPLN1 | 8.24 | 3.50 | 0.42 | 248 | 1470 | 611 | hyaluronan and proteoglycan link protein 1 |
| IGFBP3 | 24.60 | 9.89 | 0.40 | 2338 | 41497 | 16369 | insulin-like growth factor binding protein 3 |
| OXTR | 26.73 | 10.79 | 0.40 | 49 | 952 | 376 | oxytocin receptor |
| CEMIP | 44.38 | 6.80 | 0.15 | 49 | 1555 | 231 | cell migration inducing hyaluronan binding protein |

Supplementary Table 3. Primers list for RT-PCR

| Gene | Primer sequence | Product size (bp) |
| --- | --- | --- |
| MMP3 | F: tcattttggccatctcttcc  R: gtgcccatattgtgccttct | 475 |
| AKR1C1 | F: ccagagcatacatccccact  R: tccctgtcctgttttcaagg | 478 |
| SMOC1 | F: attcgtgtgaccaggagagg  R: gttgctgctattgctgtcca | 458 |
| ANGPTL2 | F: gtataacgcctgtgcccact  R: tcctgtcctgtcctggagac | 405 |
| SEMA3A | F: ttccttttggatgaggaacg  R: ctctgtcctgattgggtggt | 465 |
| TRIM15 | F: tcaaccagagcaggtgtgag  R: gagtcccatctctcccttcc | 422 |
| EGR1 | F: ctgcgacatctgtggaagaa  R: tgtcctgggagaaaaggttg | 427 |
| BMP2 | F: acttttggacaccaggttgg  R: gtggcagtaaaaggcgtgat | 409 |
| TFPI2 | F: gctgtggagggaatgacaat  R: acgaccccaagaaatgagtg | 427 |
| SFRP4 | F: cacaacggtggtggatgtaa  R: tggccttacataggctgtcc | 483 |
| SERPINE1 | F: gacatcctggaactgcccta  R: atcacttggcccatgaaaag | 467 |
| GAPDH | F: ccactggcgtcttcaccac  R: cctgcttcaccaccttcttg | 501 |


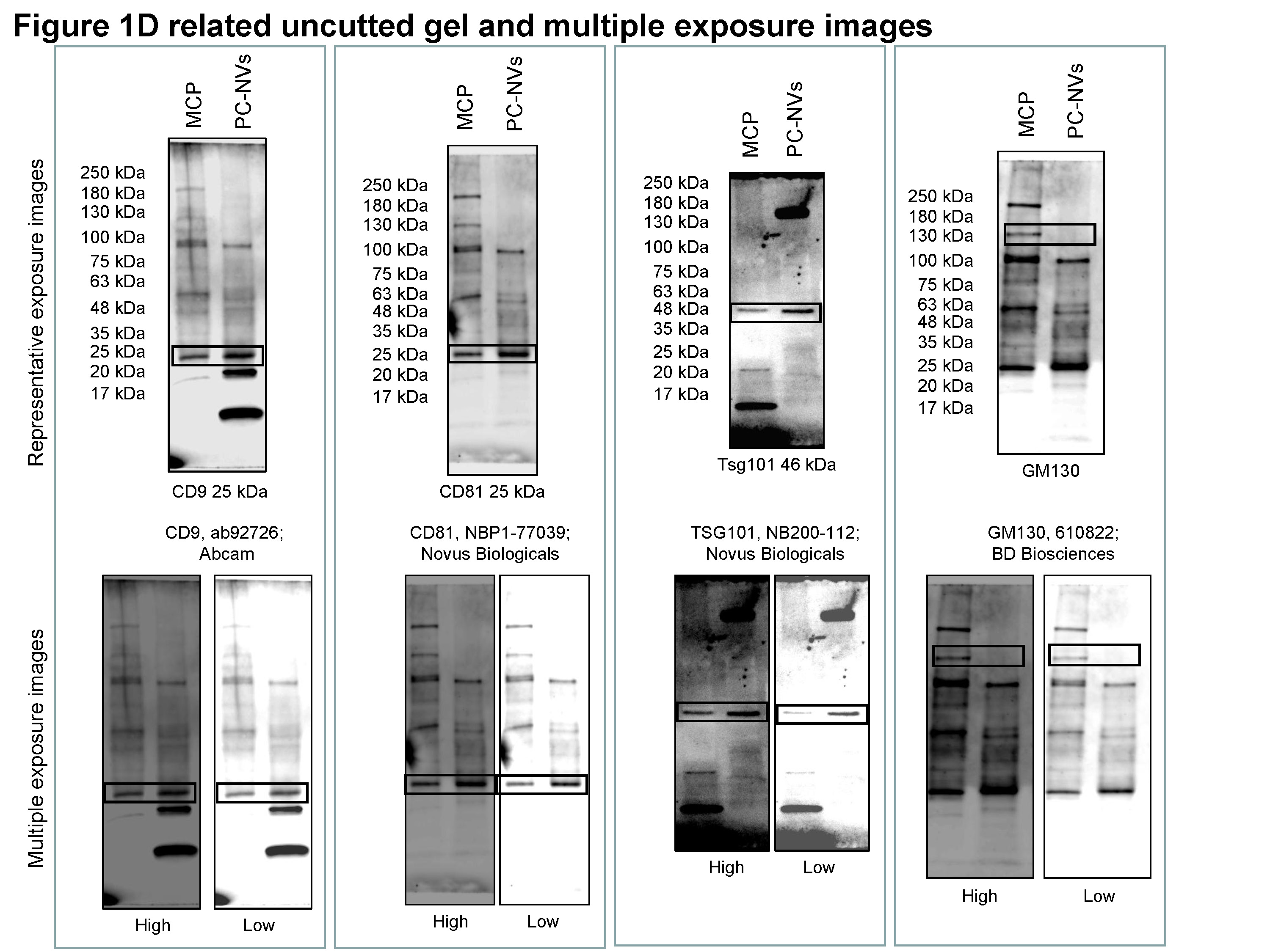


Figure 1D related un-cutted membrane multiple exposure images. Representative exposure images (top) and multiple exposure images (bottom).


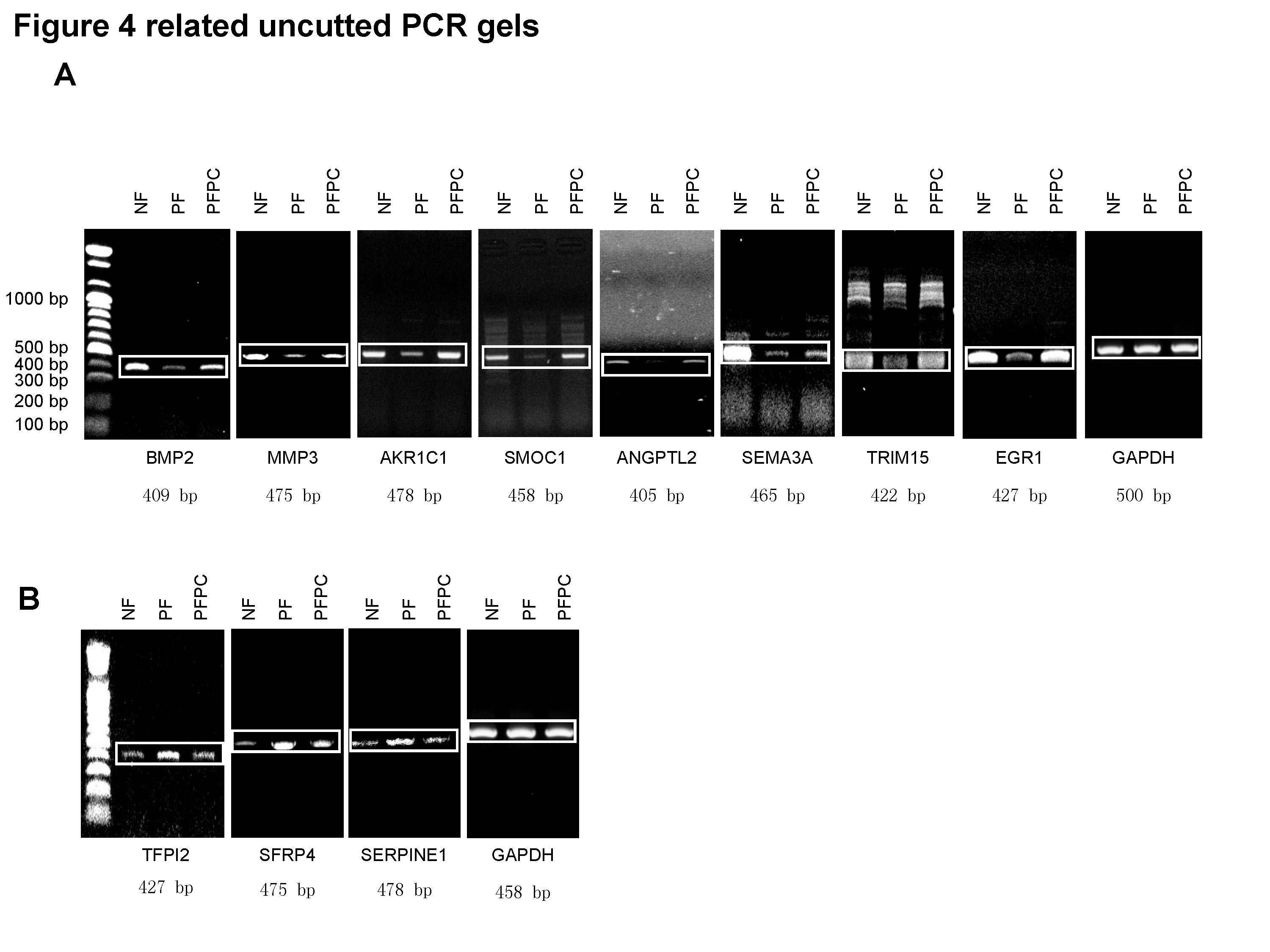


Figure 4 related un-cutted gel images.
